# New Advances in High-Entropy Alloys

**DOI:** 10.3390/e22101158

**Published:** 2020-10-15

**Authors:** Yong Zhang, Ruixuan Li

**Affiliations:** 1 Beijing Advanced Innovation Center of Materials Genome Engineering, State Key Laboratory for Advanced Metals and Materials, University of Science and Technology Beijing, Beijing 100083, China; liruixuan1208@163.com; 2 Qinghai Provincial Engineering Research Center of High Performance Light Metal Alloys and Forming, Qinghai University, Xining 810016, China; 3 Shunde Graduate School, University of Science and Technology Beijing, Foshan 528399, China

**Keywords:** high-entropy alloys, mechanical behaviors, high-entropy film, low-activation alloys

Exploring new materials is an eternal pursuit in the development of human civilization. In recent years, people have tended to adjust the order/disorder degree to explore new materials. The order/disorder can be measured by entropy, and it can be divided into two parts: the topological ordering and the chemical ordering. The former mainly refers to the ordering in the spatial configuration, e.g. amorphous alloys [1] with short-range ordering but without long-range ordering; the latter mainly refers to the ordering in the chemical occupancy, e.g. high-entropy alloys (HEAs) where components can replace each other. HEAs, in sharp contrast to traditional alloys based on one or two principal elements, have one striking characteristic: the unusually high entropy of mixing. They have not been too much noticed until a review paper entitled: ”Microstructure and properties of high entropy alloys” published in 2014 on the journal of Progress in Materials Science [2]. Lots of reports showing they exhibit five recognized performance characteristics, namely strength-plasticity trade-off breaking, irradiation tolerance, corrosion resistance, high impact toughness within a wider temperature range, and high thermal stability [3]. So far, the development of HEAs has gone through three main stages: 1. quinary equal-atomic single-phase solid solution alloys; 2. quaternary or quinary non-equal-atomic multiphase alloys; 3. medium-entropy alloys, high entropy fibers, high entropy films, lightweight HEAs, etc. Nowadays, deeper research on HEAs is urgently needed.

This Special Issue includes forty-two research articles and six review papers dedicated to the frontiers of high entropy materials, from exploring the microstructures by experiment to revealing the structure-performance relationship by simulation. These innovative studies will provide new methods to solve challenging problems and open up new possibilities for emerging research fields.

Configuration entropy plays an important role in the microstructures and properties of HEAs, which is believed to stabilize disordered solid solution phases over intermetallic compounds by lowering the Gibbs free energy. Traditionally, configuration entropy was computed by the empirical formula. In Fernández-Caballero’s work [4], a new formalism based on a hybrid combination of the Cluster Expansion Hamiltonian and Monte Carlo simulations is developed to predict the configuration entropy as a function of temperature from multi-body cluster probability in a multi-component system with arbitrary average composition. Experimentally, Haas et al. [5] used differential scanning calorimetry to determine the thermal entropy and compared it to the configurational entropy. The contributions of entropy and enthalpy to the Gibbs free energy was calculated and examined by them.

In addition to the above basic research on the entropy of the alloy system, research on HEAs mainly focuses on their composition design, preparation and processing technology, mechanical properties, and physical and chemical properties. Other main contents of this issue are listed as follows.

## 1. Compositional Design

Because HEAs are characterized by multiple components and high contents of alloying elements, the complexity of their compositional design is greatly increased compared with traditional alloys. Some empirical rules are used early on to realize simple prediction of phase structure. With the development of computer technology and simulation technology, phase diagram and first-principles methods are now widely used to help alloy design. Now, machine learning is also popular by using various algorithms. Here, our Special Issue contains three articles using phase diagram and first principles for compositional design.

A phase diagram is the geometric description of the system under a thermal equilibrium and it is the basis for the study of solidification, phase transformation, crystal growth, and solid-phase transformation. Since the 1970s, the calculation of phase diagrams (CALPHAD) based on the thermodynamic theory has become a new trend. Gorsse et al. [6] and Klaver et al. [7] conducted an in-depth study on the use of CALPHAD methods for composition design. On the other hand, Zheng et al. [8] used first principles to calculate the elastic properties of seventy different compositions in the refractory V–Mo–Nb–Ta–W system. Effects of different elements to the modulus are precisely calculated so as to optimize the composition in this refractory system.

## 2. Preparation and Processing

Traditional preparation methods of bulk HEAs mainly include vacuum arc melting, vacuum induction melting, mechanical alloying, and so on. In order to obtain a certain crystal orientation, directional solidification can also be employed. There are also some new preparation methods for bulk HEAs, such as high-gravity combustion synthesis and additive manufacturing. Chen et al. [9] reviewed the application of additive manufacturing methods in the fabrication of HEAs. Compared with the casting counterparts, HEAs prepared by additive manufacturing are found to have a superior yield strength and ductility as a consequence of the fine microstructure formed during the rapid solidification in the fabrication process. As a result, this is an effective method to improve their comprehensive properties.

Different processing methods are closely related to performance. Our Special Issue contains two research papers on the use of annealing to optimize alloys and two review papers on alloys using high pressure and welding.

Annealing is considered to be an effective method to improve the microstructure and properties of alloys, and different annealing temperature and time are closely related to comprehensive properties. Zhuang et al. [10] investigated the effect of annealing temperature on the microstructure, phase constituents, and mechanical properties of Al_0.5_CoCrFeMo_x_Ni (x = 0, 0.1, 0.2, 0.3, 0.4 and 0.5) HEAs at a fixed annealing time (10 h). They found that the alloys annealed at 80 °C showed relatively fine precipitations and microstructures and, therefore, higher hardness and yield stress. Sathiyamoorthi et al. [11] put the high-pressure torsion-processed CoCrNi alloy with a grain size of ~50 nm in different annealing conditions and they explored the optimal processing technology. The sample annealed at 700 °C for 15 min exhibited a remarkable combination of tensile strength (~1090 MPa) and strain to failure (~41%).

Pressure, as another fundamental and powerful parameter, has been introduced to the experimental study of HEAs. Many interesting reversible/irreversible phase transitions that were not expected or otherwise invisible before have been observed by applying high pressure. Zhang et al. [12] reviewed recent results in various HEAs obtained using in situ static high-pressure synchrotron radiation X-ray techniques and provided some perspectives for future research.

Welding is an important emerging area with significant potential impact to future application-oriented research and technological developments in HEAs. The selection of feasible welding processes with optimized parameters is essential to enhance the applications of HEAs. Guo et al. [13] reviewed the key recent works on welding of HEAs in detail, focusing on the research of main HEA systems when applying different welding techniques. They also highlighted the future challenges and main areas to research.

## 3. Mechanical Behaviors

Research on the mechanical properties of HEAs is the most extensive. The main research hotspots are three types of alloys: 1, 3D transitional-group-element HEAs; 2, 3D transitional-group-element HEAs with Al or Ti added; 3, refractory high-entropy alloys with excellent high-temperature properties. In addition, other trace alloying elements, such as Mo, Nb, Zr, etc., have also been added to the alloy in order to study the effect of their content on the microstructures and properties of the alloy.

HEAs based on 3D transition metals, such as CrCoNi and CrMnFeCoNi alloys, are revealed to have remarkable mechanical properties. Especially the CoCrFeMnNi alloy is famous as the Cantor alloy for its high plasticity and comparable strength. The tensile creep behavior of the CoCrFeNiMn HEA was systematically investigated by Cao et al. [14] over an intermediate temperature range (500–600 °C) and applied stress (140–400 MPa). The alloy exhibited a stress-dependent transition from a low-stress region to a high-stress region, which was characterized by the dynamic recrystallization and the grain-boundary precipitation. This alloy, deformed at high strain rates (900 to 4600 s^−1^), was investigated by Wang et al. [15]. The yield strength was sensitive to the change of high strain rates, and serration behaviors were also observed on the flow stress curves, which could be predicted by the Zerilli–Armstrong constitutive equation. Bu et al. [16] performed an in situ atomic-scale observation of deformation behaviors in a nano-scaled CoCrCuFeNi alloy. Exceptional strength was realized, and the deformation behaviors, including nano-disturbances and phase transformations, were distinct from those of corresponding bulk HEAs. The same result was confirmed in Mridha’s [17] experiments. From the perspective of theory and simulation, Ikeda et al. [18] investigated the impact of compositional fluctuations in the vicinity of stacking faults on CrCoNi and CrMnFeCoNi by employing first-principles calculations. Their research and experimental results complement and perfect each other.

Adding other alloying elements is one of the research hotspots, in order to further improve the strength. The addition of Nb promoted the precipitation of nano-phases and thus increased the strength of the alloy, and Mo addition in the CoCrFeNi alloy effectively helped to increase the corrosion resistance. Detailed work was carried out by Han et al., Wang et al., and Tsau et al. [19,20,21]. The effect of Zr addition on the CoCrFeNiMn alloy was studied by Zhang et al. [22]. They prepared the alloy by using the ZrH_2_ powders and a mechanical alloying technique. Multi-phase microstructures formed in the alloys, which can be attributed to the large lattice strain and negative enthalpy of mixing, caused by the addition of Zr. Sun et al. [23] also employed the mechanical alloying technique and prepared the CoCrNiCuZn alloy. Pd addition promoted the local- and long-range lattice distortions in CoCrFeNi alloy and also had effect on the phase stability and phase transformation. This phenomenon was revealed by Zhang et al. [24]. Tan et al. [25] then studied the effect of Mn addition on the microstructures and mechanical properties of this CoCrFeNiPd alloy.

Alloying Al and Ti in 3D transitional Co-Cr-Fe-Ni HEAs shows a strong influence on microstructure and phase composition, as well as the ability to decrease the density. They tend to strengthen the alloy by increasing the lattice distortion, as confirmed by Wu et al. [26]. Löbel et al. [27] studied the influence of Ti in the AlCoCrFeNiTi_x_ alloy, and Zhang et al. [28] characterized the partially recrystallized CoCrFeNiTi_0.2_ alloy. It seems that alloying with Ti leads to an increase in microstructural heterogeneity. Similarly aimed at the Al-Co-Cr-Fe-Ni-Ti alloy after the composition adjustment, Manzoni et al. [29] studied the microstructure and properties of the Al_10_Co_25_Cr_8_Fe_15_Ni_36_Ti_6_ alloy and Haas et al. [30] studied those of the Al_10_Co_25_Cr_8_Fe_15_Ni_36_Ti_6_ alloy. Both kinds of alloy showed beneficial particle-strengthened microstructures. Riva et al. [31] investigated the effect of Sc alloying on the Al_2_CoCrFeNi, Al_0.5_CoCrCuFeNi, and AlCoCrCu_0.5_FeNi HEAs. It caused grain refinement as well as hardness and electrical conductivity increases (up to 20% and 14%, respectively). Furthermore, the solid-state phase transformation kinetics of as-cast and cold rolling deformed Al_0.5_CoCrFeNi HEAs have been investigated by Wang et al. [32] using the thermal expansion method. Lightweight AlCrMoTi and AlCrMoTiV HEAs were designed by Kang et al. [33] and they were confirmed as solid solutions with minor ordered B2 phases. They also have superb specific hardness compared to that of commercial alloys.

With the help of their excellent mechanical properties, high-entropy thin film developed by a magnetron sputtering technique has attracted attention, which has exciting potential to make small-structure devices and precision instruments with sizes ranging from nanometers to micrometers. Liao et al. [34] fabricated Al_0.3_CoCrFeNi film, and Zhang et al. [35] prepared (Al_0.5_CrFeNiTi_0.25_)N_x_ high-entropy films. It showed that the phase structure changes from the amorphous to the face-centered cubie (FCC) structure with increasing N content, which was proved to be related with the atomic size difference in the alloy system. Similarly, (AlCrTiZrV)Si_x_N high entropy film was prepared and characterized by Niu et al. [36]. Then, an Al_0.5_CoCrCuFeNi coating was successfully prepared on an AZ91D magnesium alloy surface [37], and an AlCoCrFeNi coating was prepared on the stainless steel substrate [38]. The coating obviously had significant high hardness, high wear resistance, and corrosion resistance, and can integrate the properties of the substrate.

The development and preparation of composites have realized the complementary advantages of a variety of different component materials, which can further improve the overall performance of alloys. Zhang et al. [39] prepared a high-entropy ceramic composite by using HfC, Mo_2_C, TaC, and TiC in pulsed-current processing. Furthermore, Li et al. [40] used a mixture of carbides and oxides in the preparation of an NbMoCrTiAl HEA. With the presence of approximately 7.0 vol. % Al_2_O_3_ and 32.2 vol. % TiC-reinforced particles, the compressive fracture strength of the composite reached 1542 MPa, and this was increased by approximately 50% compared with that of the as-cast NbMoCrTiAl HEA. Furthermore, two types of novel HEA/diamond composites were reported, providing a new model for material development [41,42].

It can be found that nano-precipitation phases appear widely in HEAs, which play a vital role in improving their strength and plasticity. In this case, Wang et al. [43] summarized precipitation behavior and precipitation strengthening in HEAs comprehensively, including the morphology evolution of second-phase particles and precipitation strengthening mechanisms. They claimed that the challenge in the future is to design a stable, coherent microstructure in different solid-solution matrices.

Besides room-temperature mechanical properties, HEAs tend to exhibit great high-temperature properties for their high-entropy stabilization effect. The Hf-Nb-Ta-Ti-Zr refractory HEA shows great high-temperature properties and room-temperature properties. Zýka et al. [44] presented investigations of the room-temperature tensile mechanical properties of selected three- and four-element medium entropy alloys derived from the Hf-Nb-Ta-Ti-Zr system, and they found that it is the five-element HEA alloy that exhibits the best combination of strength and elongation. Tseng et al. [45] aimed to reveal the effects of Mo, Nb, Ta, Ti, and Zr on mechanical properties of equiatomic Hf-Mo-Nb-Ta-Ti-Zr alloys. Another VCrFeTa_x_W_x_ HEA, which has great high-temperature properties and excellent heat-softening resistance, was studied and recommended by Zhang et al. [46].

## 4. Physical and Chemical Properties

In addition to mechanical properties, research on the physical and chemical properties of HEAs mainly focuses on soft magnetic properties, radiation resistance, electrical properties, and corrosion resistance.

Because HEAs contain more principal elements, including magnetic elements, such as Fe, Co, Ni, and Mn, and these magnetic elements are mixed with other elements, HEAs exhibit different magnetic properties compared to traditional alloys. Li et al. [47] exhibited the effects of Sn addition on the soft magnetic properties of dual-phase FeCoNi(CuAl)_0.8_Sn_x_ (0 ≤ x ≤ 0.10) HEAs. They showed that saturation magnetization of the alloy increased greatly, while the remanence (Br) decreased after the addition of Sn. Furthermore, thermomagnetic curves indicated that the phases of the alloy will transform from FCC with low Curie temperatures (Tc) to the body-centered cubic (BCC) phase with high Tc at temperatures of 600–700 K. For the FeSiBAlNi alloy, the effects of Co and Gd addition combined with subsequent annealing on microstructures were investigated by Zhai et al. [48]. FeSiBAlNi-based HEAs also possessed soft magnetism, especially the as-annealed FeSiBAlNiGd alloy, which can be ascribed to the formation of Gd-oxides.

Some HEA systems also have good radiation resistance. Disordered solid-solution phase structure is mainly formed in HEAs. The biggest feature of their structure is the large lattice distortion caused by the difference in atomic size and the high configuration entropy. Therefore, atomic-level stress may be formed. In addition, the sluggish diffusion effect is caused by the interaction of multiple components. As a result, HEAs have shown excellent performance in radiation resistance, which provides new ideas for the development of nuclear materials. CoCrFeCuNi alloys were irradiated with a 100 keV He^+^ ion beam by Wang et al. [49]. It was indicated that Cu-rich phases were favorable sites for the nucleation and gathering of He bubbles. At ion doses of 2.5 × 10^17^ ions/cm^2^ and 5.0 × 10^17^ ions/cm^2^, the HEAs showed obvious hardening, which could be attributed to the formation of large amounts of irradiation defects.

## 5. Outlook

The development of HEAs has only been occurring for no more than ten years until now, and people’s understanding of this material is still in its infancy. This Special Issue summarizes some of the latest developments in HEAs. Even though not all the questions related to HEAs have been given a reasonable explanation in this issue, it does pay attention to some typical research hotspots, and significant progress can, indeed, be seen. We believe this special issue will guide further research and promote the property breakthrough of HEAs.
